# Plant Protein *O*-Arabinosylation

**DOI:** 10.3389/fpls.2021.645219

**Published:** 2021-03-18

**Authors:** Bent Larsen Petersen, Cora A. MacAlister, Peter Ulvskov

**Affiliations:** ^1^Department of Plant and Environmental Sciences, Faculty of Science, University of Copenhagen, Copenhagen, Denmark; ^2^Department of Molecular, Cellular and Developmental Biology, University of Michigan, Ann Arbor, MI, United States

**Keywords:** plant protein O-glycosylation, hydroxyproline-arabinosylation, secretory pathway, extensin, peptide hormone, plant allergens, arabinogalactan protein, hydroxyproline glycoprotein module

## Abstract

A wide range of proteins with diverse functions in development, defense, and stress responses are *O*-arabinosylated at hydroxyprolines (Hyps) within distinct amino acid motifs of continuous stretches of Hyps, as found in the structural cell wall extensins, or at non-continuous Hyps as, for example, found in small peptide hormones and a variety of plasma membrane proteins involved in signaling. Plant *O*-glycosylation relies on hydroxylation of Prolines to Hyps in the protein backbone, mediated by prolyl-4-hydroxylase (P4H) which is followed by *O*-glycosylation of the Hyp C_4_-OH group by either galactosyltransferases (GalTs) or arabinofuranosyltranferases (Ara*f*Ts) yielding either Hyp-galactosylation or Hyp-arabinosylation. A subset of the P4H enzymes with putative preference to hydroxylation of continuous prolines and presumably all Ara*f*T enzymes needed for synthesis of the substituted arabinose chains of one to four arabinose units, have been identified and functionally characterized. Truncated root-hair phenotype is one common denominator of mutants of Hyp formation and Hyp-arabinosylation glycogenes, which act on diverse groups of *O*-glycosylated proteins, e.g., the small peptide hormones and cell wall extensins. Dissection of different substrate derived effects may not be regularly feasible and thus complicate translation from genotype to phenotype. Recently, lack of proper arabinosylation on arabinosylated proteins has been shown to influence their transport/fate in the secretory pathway, hinting to an additional layer of functionality of *O*-arabinosylation. Here, we provide an update on the prevalence and types of *O*-arabinosylated proteins and the enzymatic machinery responsible for their modifications.

## Introduction

Glycosylation of proteins is a common post-translational modification (PTM) on a large number of proteins across the domains of life. While some types of glycosylation, at least in part, are conserved broadly, others display more limited phylogenetic distributions. Specialization is particularly prominent for *O*-linked modifications where sugars are added to the oxygen present on mainly serine and threonine, or in the case of plants, hydroxyproline (Hyp). Hyp *O*-glycosylation takes place in the secretory pathway and occurs in two major forms, Hyp *O*-galactosylation and Hyp *O*-arabinosylation. Hyp-arabinosylation was first discovered in the structural cell wall glycoprotein family extensins (Lamport, 1963). It has since then been found in a number of unrelated families of proteins including small peptide hormones and (receptor) kinases. This review provides an update on recent insights in *O*-arabinosylation prevalence, functionality, and regulation. Some emphasis will be put on the role of glycosylation in correct protein processing and targeting that takes place in the secretory pathway. The importance of arabinosylation to cell wall sensing and signaling will also be covered.

Hyp-*O*-arabinosylation radically sets plant *O*-glycosylation apart from that of mammalian cells. In fact, a number of allergies in man are caused by plant allergens that feature Hyp-*O*-arabinosylation. This has implications: firstly, the prospect of using mammalian cells as a clean slate system for constructing plant *O*-arabinosylation in a host cell; secondly, spurious Hyp-*O*-arabinosylation of therapeutic human proteins when expressed in plants must be prevented for plant cells to become useful cell factories for therapeutic proteins (Gomord et al., 2010).

## HYP-*O*-Glycosylated Proteins

### Contiguous Hyp-*O*-Arabinosylation

**Figure 1:**
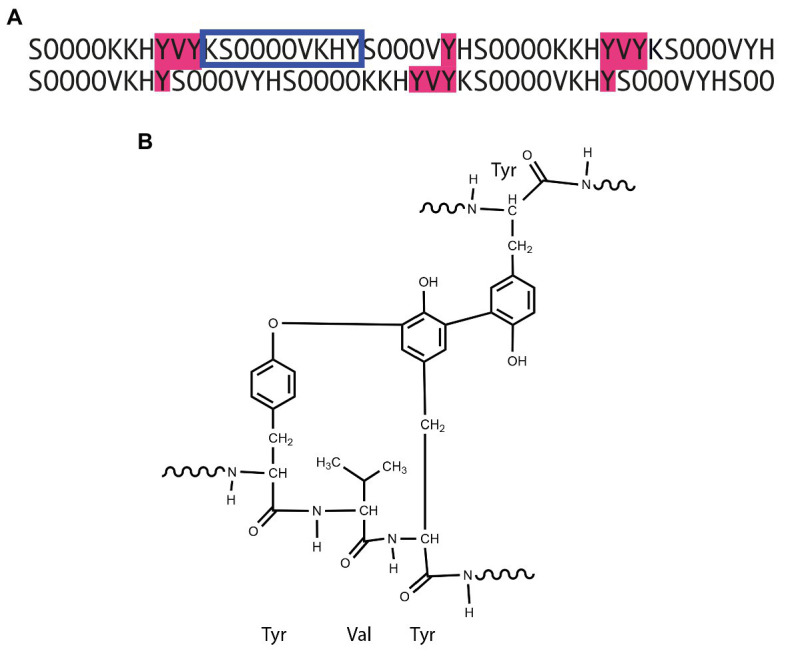
Interchain phenolic cross-links in extensin. **(A)** Staggered partial alignment of *Arabidopsis* EXT3 with potential pulcherosine cross-links shaded in pink. **(B)** Chemical structure of the pulcherosine cross-link. The blue frame in **(A)** indicates the smallest extensin motif with experimentally validated arabinosylation (Beuder et al., 2020).

**Figure 2:**
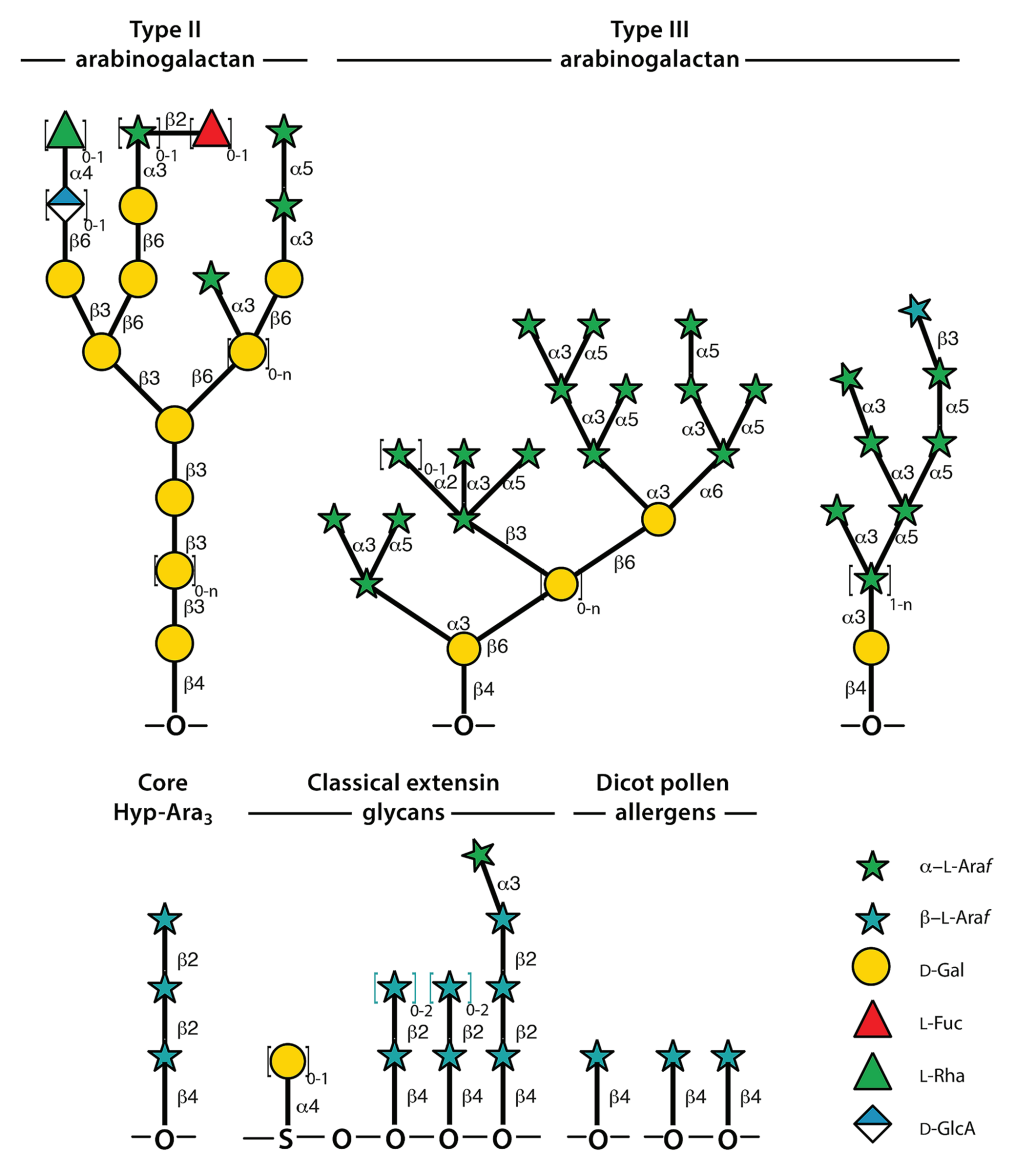
Representative structures of Hyp-glycans. Some of the structural variation is indicated by enclosing repeat structures in square brackets followed by indices representing the range of replicates, where *n* means that the maximum is not known. Not all structural variations of arabinogalactans are shown. The representative Type-II structures were assembled from Tryfona et al. (2012) and Seifert (2020). Type-III structures and the dicot pollen allergens were derived from Leonard et al. (2005) and Leonard et al. (2010). The ordering of arabinoside side-chains in extensins is not known; hence, the choice of placing Hyp-Ara*f*_4_ toward the C-terminus of the motif is arbitrary. The structures shown are from Kieliszewski (2001).

**Contiguous Hyp-*O*-arabinosylation** refers to the class of glycosylation motifs originally discovered in extensins, see Figure 1A, and demonstrated to lead to serine α-galactosylation and core arabinosylation of Hyps with β-1,2-linked arabinofuranosides (Ara*f*) of length 1-3 (Shpak et al., 1999; Kieliszewski, 2001). “Contiguous” alludes to the characteristic repetitive Ser-Hyp_3+_ that defines extensins. Hyp-Ara*f*_3_ may be further elongated with an α-linked Ara*f* and occasionally a fifth arabinosyl residue of unknown regio- and stereochemistry (Mazau and Esquerretugaye, 1986; Møller et al., 2017). Arabinoside profiles are characteristic of a species (Lamport and Miller, 1971), yet vary between tissues (Estevez et al., 2006; Møller et al., 2017) and during development as recently shown in a study of developing cotton fiber cells (Guo et al., 2019). The glycosylation machinery, which we will return to below, is ancient at least considering the three β-linked residues that we refer to as the core structure (Figure 2), whereas the genes encoding the extensin polypeptides appear to be streptophyte inventions (MacAlister et al., 2016; Møller et al., 2017) with considerable structural variation across the plant kingdom; particularly with notable differences between grasses and other flowering plants (Carpita, 1996). Classical extensins feature a cross-linking motif comprising two Tyr residues that may be coupled oxidatively, both intra- and inter-chain (Mnich et al., 2020). The most important inter-chain cross-link is pulcherosine (Figure 1). The YVY cross-linking motif common to canonical EXTs (Figure 1) appears to be present in commelinid monocot leucine-rich repeat extensins (LRXs) but missing in the extensins of this group. Arabinoxylan-linked ferulic and p-coumaric acids have been proposed to replace extensins as cross linkers in these plants (Francoz et al., 2015).

Extensins play roles in cell wall architecture (Hijazi et al., 2014). They are basic proteins that interact with acidic pectic polymers (Cannon et al., 2008). *In vitro* studies suggest that proper glycosylation of extensins is required for acquiring the rod-like extended structures which are required for sequestering cell wall polymers and extensin cross-linking (see Figure 1; Vanholst and Varner, 1984; Stafstrom and Staehelin, 1986). A hypothesis has been presented for their mode of insertion by reptation into the wall orthogonally to the plasma membrane (Kieliszewski et al., 2011). Extensins are used for wall repair, e.g., following pathogen attack (Showalter et al., 1991) but are also secreted in mucilage to play roles in non-pathogenic microbial interactions (Castilleux et al., 2018) and during cell plate formation.

Extensin cross-linking is catalyzed by peroxidases (Schnabelrauch et al., 1996) and positioning of the flanking Tyr residues, as well as the glycan structure, are integral to the process: the importance of extensin *O*-glycosylation (Gille et al., 2009), and in particular the fourth Ara*f* residue, for cross-linking has been substantiated *via in vitro* studies, which demonstrated that the initial rate of cross-linking was primarily determined by the number of cross-linking motifs (Figure 1) in the protein backbone and by Hyp-Ara*f*_4_ (Chen et al., 2015). Complete removal of the fourth Ara*f* through knockout of the *Extensin Arabinose Deficient* (*ExAD*) gene in *Arabidopsis*, however, displayed no visible phenotypic effects (Møller et al., 2017). Actual cross-linking at the molecular level was not examined.

Schnabelrauch et al. (1996) showed that de-glycosylated extensin monomers were not cross-linked *in vitro* by extensin peroxidase from tomato cell culture medium substantiating the role for arabinosylation in extensin self-assembly and cross-linking. Recently, two extensin peroxidases have been identified and demonstrated to be essential to tapetum and pollen development (Jacobowitz et al., 2019). Interestingly, the two enzymes belong to the related F and C clades of peroxidases and are not very similar to previously known extensin peroxidases from *Vitis* and tomato (M clade), and French bean and lupin (A clade) (Mnich et al., 2020).

Covalent linkages between the different polymer classes, hemicelluloses, pectins, and hydroxyproline-rich glycoproteins (HRGP), were central to the earliest cell wall models (Keegstra et al., 1973), and then less appreciated (Talbott and Ray, 1992; Carpita and Gibeaut, 1993) but growing evidence for the occurrence of covalent cross-links between polymers has to be taken into account in cell wall analysis (Fry, 2011). Cross-links between pectic polymers and extensin have been demonstrated in sugarbeet (Nunez et al., 2009) and cotton (Qi et al., 1995) but the type of linkage is unknown.

Arabinosylation of extensins is essential for root hair integrity and the root hair phenotypes played a key role in the characterization of the arabinosylation mutants (Velasquez et al., 2011), see below. Whether similar defects occur in less easily observable cells featuring diffuse growth is an open question. Generalizing from root hairs to the larger class of tip growing cells should be done with caution as their cell wall compositions differ considerably. Root hairs contain high levels of cellulose, while pollen tubes produce little cellulose, but abundant callose (Park et al., 2011; Akkerman et al., 2012; Chebli et al., 2012). Root hairs also contain an acidic xyloglucan that is important for normal development (Pena et al., 2012) that does not occur in pollen tubes (Dardelle et al., 2010). In general, xyloglucan mutants show root hair growth defects, while pollen tubes appear to be unaffected (Cavalier et al., 2008), even though pollen tubes do contain xyloglucan (Lampugnani et al., 2013).

Despite the differences in their cell wall organization (Chebli et al., 2012), pollen tubes are also dependent on Hyp-arabinosylation (Ogawa-Ohnishi et al., 2013; MacAlister et al., 2016). Double mutants of *Arabidopsis hpat1* and *hpat3* (Hyp-arabinosyltransferases, see below) display severe male fertility defects due to compromised pollen tube cell wall polarity (Beuder et al., 2020). A recent genetic screen for suppressors of the *hpat* pollen phenotype identified mutants in the late stages of the secretory pathway, specifically in the vesicle-tethering exocyst complex. Beuder et al. (2020) found that *hpat* pollen tubes had increased rates of HPAT-modified protein secretion compared to WT pollen tubes and the rate of secretion was reduced in the suppressed line to near WT levels. This observation is consistent with a “toxic” effect of increased secretion of un-arabinosylated proteins. Which specific HPAT-target protein(s) may be responsible is unknown, but candidates include the canonical extensins or other proteins carrying Hyp-*O*-arabinosylation sites (see below).

Extensins are members of the HRGP superfamily which also comprises the lightly glycosylated proline-rich proteins (PRPs) and the often very heavily glycosylated arabinogalactan proteins (AGPs, see Figure 2). Early studies revealed that AGPs to be differentially and transiently expressed during embryogenesis in oilseed rape (Pennell et al., 1991) and are also implicated in somatic embryogenesis (Kreuger and Vanholst, 1993) suggesting roles in differentiation, cell identity, and cell-cell interaction. Examination of AGPs in carrot root similarly points to roles in differentiation (Knox et al., 1991). The glycan structure of AGPs is important as documented by the requirement of a fucosylated AGP for root cell expansion (van Hengel and Roberts, 2002). AGPs are important to growth of pollen tubes and more precisely in the endosomal transport that is essential to tip growth, reviewed in Dehors et al. (2019). Glycosylation sites in AGPs are of the clustered **non-contiguous Hyp *O*-galactosylation** type. The glycans are β-1,3-galactans featuring β-1,6-linked galactan side-chains and further decorated with rhamnose, (Me-)glucuronic acid, arabinose, and fucose (Figure 2; Tryfona et al., 2012). AGPs were reviewed recently (Seifert, 2020). One of the subfamilies, referred to as *hybrid* in a newly updated HRGP classification scheme (Liu et al., 2020), comprises both sites for clustered **non-contiguous Hyp *O*-galactosylation** and sites for **contiguous Hyp *O*-arabinosylation** and is thus included here.

While hybrid HRGPs refer to proteins featuring domains from more than one HRGP sub-family, chimeric HRGPs comprise one type of HRGP domain plus domains from another gene family (Liu et al., 2020). A wide variety of protein families fall into this class.

The updated HRGP classification comprises the following sub-classes of chimeric proteins with extensin domains: LRXs, proline-rich extensin-like receptor kinases (PERKs), formin-homolog EXTs (FH EXTs), and other chimeric EXTs.

The *Arabidopsis* genome encodes four LRXs involved in pollen and pollen tube development and seven that are expressed in vegetative tissues (Fabrice et al., 2018). LRX proteins function *via* the binding of their LRR domain to rapid alkalinization factors (RALF) signaling peptides and *Catharanthus roseus* receptor-like kinase1-like (CrRLK1L) proteins to monitor cell wall integrity (Blackburn et al., 2020; Herger et al., 2020; Moussu et al., 2020). The EXT-like domain of *Arabidopsis* LRX1 is required for its insolubilization in the cell wall and is essential for its function in root hair elongation (Ringli, 2010).

The molecular function of the PERK family is less well understood. *AtPERK4* is involved in the response to the plant hormone abscisic acid and its kinase activity is activated by abscisic acid and calcium (Bai et al., 2009). Many PERKs are expressed primarily or exclusively in pollen and pollen tubes, but not all PERKs play roles in pollen development and in cell wall sensing (Chen et al., 2020), i.e., monitoring cell wall stresses during cell expansion, for example, but roles in responding to wounding and pathogens are also documented (Qanmber et al., 2019).

The intercellular part of formin-homolog EXTs interacts with actin and is thus another good candidate for coordinating the cell wall to the cell interior (Borassi et al., 2016). The EXT-like extracellular domain of *Arabidopsis* FH1 has been shown to physically interact with the cell wall, limiting its lateral mobility at the plasma membrane (Martiniere et al., 2011). Other members of this family are required for proper root hair and pollen tube elongation (Cheung et al., 2010; Huang et al., 2013; Lan et al., 2018), suggesting a possible role in coordinating a polarized actin cytoskeleton and a polarized cell wall during tip growth.

It is broadly accepted that the occurrence of the SP_3+_ motif is sufficient for Pro hydroxylation and *O*-arabinosylation but glycosylation of these domains in EXT chimeras has not been validated experimentally as far as we are aware.

Solanaceous lectins fall in the category of *other chimeric*. They feature chitin-binding domains interspaced with heavily glycosylated extensin-like regions (Kieliszewski et al., 1994). Roles in pathogen defense are inferred from the chitin affinity and in a few cases also documented (Chen et al., 2018). Their evolutionary origin is a conundrum: tomato (Oguri et al., 2008) and potato lectin (Van Damme et al., 2004) are closely related but their domain organization is quite different. Smaller differences in lectin specificity are inferred from *in silico* analyses (Jain et al., 2020).

Finally, two pollen allergens are considered which also fall in the category of *other chimeric* due to the presence of a defensin domain. Defensins are evolutionary ancient peptides involved in innate immunity. These allergens are also hybrid as they feature both **non-contiguous Hyp *O*-galactosylation** and sites for **contiguous Hyp *O*-arabinosylation**. The structures to be discussed are shown in Figure 2. Mugwort, *Artemisia vulgaris* Art v 1 (Himly et al., 2002), and short ragweed, *Ambrosia artemisiifolia* amb a 4 (Leonard et al., 2010), are both modular proteins featuring an N-terminal defensin-like and C-terminal hydroxyproline-rich domain. C-terminal SP_3_ motifs in Art v 1 are β-arabinosylated, often on adjacent Hyps while some non-contiguous Hyps carry Type-III arabinogalactans (see Figure 2; Leonard et al., 2005). Type III arabinogalactans feature a galactan backbone that carry branched α-linked arabinans. The backbone is β-1,6-linked in contrast to Type-II arabinogalactans (Figure 2) of standard AGPs, yet, it is Yariv binding. If the Hyp-Ara*f*_1_ side-chains are synthesized as such, it raises the question how they evade being elongated. Processing is an alternative (Guo et al., 2019), but while β-arabinofuranoside degradation is known from bacteria (Miyake et al., 2020; Saito et al., 2020), it remains to be discovered in plants. The ragweed allergen is related but differs in that the P_3_ motifs are preceded by an Ala rather than Ser, yet, the Hyps carry single, β-linked Ara*f* just as the mugwort allergen. The Type III arabinogalactans are also different in that the galactan backbone is of length 1 and some of the α-arabinan side-chains are capped with a β-linked Ara*f*. If α-arabinan capping is more wide-spread, and then the Ray1 gene (Gille et al., 2013), characterized in *Arabidopsis*, may encode a candidate GT for transferring the terminal β-linked Ara*f*.

### Non-contiguous Hyp *O*-Arabinosylation

**Figure 3:**
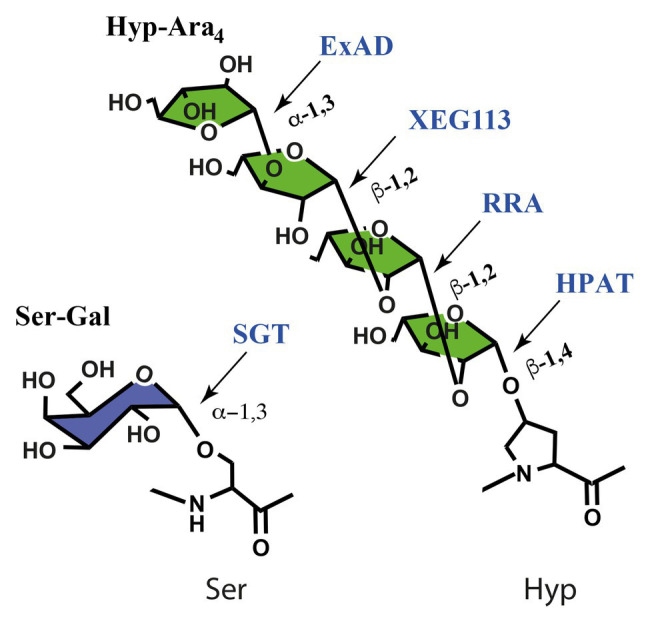
Glycosyltransferases involved in serine galactosylation and in Hyp arabinosylation, named after their mutant phenotype in *Arabidopsis* or screen in which the GT was identified (*At*XEG113). *At*SGT and *At*ExAD are specific to extensins, while the β-Ara*f*Ts have a wide selection of substrates. SGT, serine galactosyltransferase; HPAT1-3, hydroxyproline arbinosyltransferase 1-3; RRA1-3, reduced residual arabinose 1-3; XEG113, xyloglucan endoglucanase 113; ExAD, extensin deficient arabinose. The ortholog to *At*XEG113 in tomato is the *fasciated and branched* 2, *fab2*, mutant (Xu et al., 2015).

Non-contiguous Hyp *O*-arabinosylation refers to synthesis of the Ara*f*_3_ core structure onto Hyps that are not derived from the typical SP_3+_ motif. The core structure is never elongated with a fourth α-linked Ara*f* (Figure 3). Hyp-Ara*f*_3_ is found on a number of diverse proteins.

A number of small peptide hormones and certain other allergens fall under this heading. Genome and transcriptome analyses have identified more than 1,000 potential peptide hormones in *Arabidopsis* (Lease and Walker, 2006; Matsubayashi, 2014). Several PTMs are regularly required for peptide maturation and function. Most peptide hormones thus undergo PTM modifications and hydroxyproline arabinosylation and sulfation of tyrosine residues, for example, has been documented in a few instances. CLAVATA3 (CLV3) is part of a network involved in stem cell maintenance and differentiation in meristems (Clark et al., 1996; Schoof et al., 2000) and it features a single Hyp-Ara*f*_3_ side-chain (Ohyama et al., 2009). Loss of CLV3 results in disruption of the meristem size control pathway leading to a progressive increase in meristem size referred to as fasciation (Clark et al., 1995). In one study in tomato where the initiating and elongating arabinosyltransferases acting on CLV3 were knocked out by gene editing, the most severe fasciation resulted from the knockout of the initiating arabinosyltransferase, i.e., plants lacking the entire tri-arabinoside (Xu et al., 2015). The phenotype could only be rescued by arabinosylated CLV3, demonstrating that arabinosylation of CLV3 is important for its function. “Weaker” meristem phenotypes were observed in mutants with truncated arabinosides, indicating that CLV3 must be fully arabinosylated for full activity (Xu et al., 2015). This conclusion is corroborated by *in vitro* studies using chemically synthesized differentially arabinosylated CLV3 peptides. CLV3/EMBRYO SURROUNDING REGION-related (CLE) peptides are related to CLV3 and several CLEs can complement *clv3* knockouts (Ni and Clark, 2006) and for CLE2 Hyp *O*-arabinosylation was demonstrated and also that it is essential to function (Ohyama et al., 2009).

The small peptide hormone PLANT PEPTIDE CONTAINING SULFATED TYROSINE 1 (PSY1) is a growth-promoting secreted tyrosine-sulfated glycopeptide whose receptor directly phosphorylates and activates the plasma membrane H^+^-ATPase, which results in acidification of the apoplastic space facilitating cell wall loosening and is, therefore, a key step in cell expansion (Fuglsang et al., 2014; Mahmood et al., 2014). The mature PSY1 features a sulfated Tyr and a single Hyp-Ara*f*_3_ side-chain (Amano et al., 2007).

C-TERMINALLY ENCODED PEPTIDE (CEP) is Hyp-glycosylated at Asp-Ser-**Hyp**-Gly-Val (Patel et al., 2018), CLV3 and CLE2 share Gly-**Hyp**-Asp-Pro as glycosylation site while PSY1 is glycosylated at the first Hyp of Val-**Hyp**-Hyp-Ser. A large data-set is required to define a common motif or a set of motifs that lead non-contiguous Hyp *O*-arabinosylation. Structural features and biological functions of the small peptide hormones have been reviewed extensively (Matsubayashi, 2011, 2014; Tavormina et al., 2015; Stuhrwohldt and Schaller, 2019).

A number of pollen allergens belong under this heading but differ from the peptide hormones in two respects: arabinosylation is not confined to the core structure, Hyp-Ara*f*_3_; shorter side-chains are also found but not Hyp-Ara*f*_4_. The occurrence of a β-expansin domain in some of these suggests roles in pollen-pistil interactions (Wang et al., 2016). Major allergens from ryegrass and Timothy grass, *Lolium perenne* 1 (Lol p 1) and *Phleum pretense* 1 (Phl p 1), are β-expansins (Li et al., 2003) while Phl p 5 features a pollen RNase domain (Bufe et al., 1995). Phl p 1 and 5 were experimentally shown to carry Hyp and Hyp-Ara*f*_1-3_ substitutions (Halim et al., 2015) and Lol p 1, a close homolog of Phl p 1, was shown to carry Hyp and likely Hyp substituted arabinosylation (Li et al., 2003).

## Biosynthetic Machinery

The arabinosyltransferases (Ara*f*Ts) that catalyze Hyp-glycosylation are encoded by single genes or small gene families. There are three Ara*f*Ts in *Arabidopsis* that transfer the first and three that transfer the second β-linked Ara*f* and only one Ara*f*T for each of the third and fourth Ara*f*, see Figure 3. This means that the diverse gene families considered above largely share the same glycosylation machinery. This raises questions regarding how Hyp-Ara_1_ in allergens and Hyp-Ara_3_ in peptide hormones evade further elongation; and it raises questions as to the recognition of glycosylation motifs. For the latter question the prolyl-4-hydroxylases (P4Hs), which insert the C_4_-OH group onto prolyl residues in the protein backbone, thus enabling recognition of appropriate Hyp glycosylation sites for the initiating GTs should be considered. The P4Hs are type II membrane anchored proteins and form a family with 13 members in *Arabidopsis*. Some insights in the labor division, i.e., substrate specificities, among the P4Hs have been documented. Consecutive Pro residues appear to be a favored motif for some P4Hs, and AtP4H5, -2, and -13 have been identified as involved in proline hydroxylation of cell wall extensins *in vivo* (Velasquez et al., 2011, 2015b). However, the widespread prevalence of Hyp residues without glycosylation necessitates that the initiating GTs also play roles in recognition of arabinosylation motifs.

Ara*f*Ts, both the enzymes that target the Hyps in the peptide backbone and those that elongate the glycan chains are Golgi localized type II membrane anchored proteins. The three hydroxyproline *O*-arabinosyltransferases, HPAT1-3 are founding members of CaZY-family GT95, identified and functionally characterized by Ogawa-Ohnishi et al. (2013). Family GT95 is related to family GT96 to which the extensin serine α-galactosyltransferase SGT1 belongs (Saito et al., 2014). Mutant data showed that HPAT1, HPAT2, and HPAT3 redundantly contribute to arabinosylation of the abundant extensin 3, EXT3, in *Arabidopsis*, and that at least subgroups of the CLE peptides are mainly arabinosylated by HPAT3. The three enzymes in *Arabidopsis* thus have overlapping but distinct target substrate preferences (Ogawa-Ohnishi et al., 2013). It would be tempting to guess that the role of the Ser-galactosylation would be to guide either the P4Hs or the arabinosyltransferases but the *sgt-1* knockout mutant is unaffected in arabinosylation (Møller et al., 2017).

The β-arabinosyltransferases that add the second residue, reduced residual arabinose 1-3 (RRA1-3; Egelund et al., 2007; Velasquez et al., 2011), and the third residue, XEG113 (Gille et al., 2009), are all GT77 family proteins, see Figure 3, and are conserved over large phylogenetic distances. We have detected Hyp-Ara*f*_3_ in the prasinophyte *Ostreococcus tauri* (unpublished observation), as well as gene orthologues in sequenced Chlorophytes but not in Rhodophyte genomes (Ulvskov et al., 2013). Extensin arabinose deficient (ExAD; Møller et al., 2017) belongs to family GT47 and is responsible for transferring the fourth α-linked arabinosyl residue, see Figure 3. ExAD is probably neofunctionalised in streptophytes, as probable homologs exist in chlorophytes which do not feature the fourth Ara*f* (Møller et al., 2017).

## Discussion: Outstanding Questions and Future Research in *O*-Arabinosylation

### Hyp O-Glycosylation Prevalence in Plants

Non-contiguous Hyp *O*-arabinosylation appears to be present on various proteins encoded by several gene families. The prevalence of this PTM is probably significant and its detection dependent on whether or how these PTMs were included in the glycoprotein analysis. The *O*-glycoproteome is likely to be markedly under-annotated especially with respect to number of glycosylation sites and perhaps also with respect to described structures (for some indications of the latter, see, for example, Leonard et al., 2005, 2010; Halim et al., 2015). Some initial strategies for identification of proteins prone to Hyp-glycosylation in intrinsically disordered proteins (IDPs) to which the HRGPs belong have been provided (Johnson et al., 2017), but general high throughput strategies have not been attempted. Recently high throughput system approaches, based on lectin glycopeptide/glycan enrichment and advanced Mass spec site specific glycosylation analysis, for assessing glycosylation in the mammalian proteome, have resulted in multiple doublings of the glycosylation sites, glycosylated proteins, and structures (reviewed in Schjoldager et al., 2020 and refs herein). For mammals, it is estimated that the vast majority of secretory proteins (>85%) are glycosylated (Zielinska et al., 2010; Steentoft et al., 2013) and most nuclear and cytoplasmic proteins undergo dynamic *O*-GlcNAcylation (Hart, 2019). Implementation of such strategies on the plant Hyp *O*-glycome is expected to yield similar insights, at least.

### Protein Complexes in Biosynthesis

The sugar donor substrate for arabinosylation exists as UDP-Ara*p* and thus requires a sugar ring contraction to produce UDP-Ara*f* and eventually the structures discussed above. This reaction is catalyzed by neofunctionalized GT75s (Konishi et al., 2007). These proteins are cytosolic but associated with the Golgi membrane (Dhugga et al., 1991), while the GTs are located in the secretory pathway. This calls for organization of the mutase, a transporter and the Ara*f*Ts on the luminal side of the Golgi membrane as analyzed in detail recently (Saqib et al., 2019).

There are also indications of protein complexes playing a role in Pro hydroxylation. It has been demonstrated that *At*P4H5 may form homodimers and heterodimers with *At*P4H2 and *At*P4H13 in the Golgi and that P4Hs complexation may be required for prober localization and Pro hydroxylation (Velasquez et al., 2015a). Further studies are needed to validate these findings and potential complex-formation between initiating P4H and Hyp-glycosyltransferases though the reverse genetics and/or advanced protein-protein interaction techniques may be hampered by the high prevalence of isoenzyme redundancies *in vivo*. Defining sites for **non-contiguous Hyp *O*-arabinosylation** is a major research challenge and we expect that many more families of secreted proteins will be found to carry arabinosides.

### Shared Glycosylation Pathways: Genotype to Phenotype

The degree to which the general Hyp-arabinosylation machinery (i.e., the P4Hs and Ara*f*Ts required for Hyp-arabinosylation) are shared between the classes of target proteins (i.e., contiguous and non-contiguous proline hydroxylation and Hyp-arabinosylation) is unclear. But, some cases of substrate preference have been reported. For example, when the CLE2 peptide was overexpressed in *Arabidopsis hpat3-1* mutants, the peptide was largely detected in an un-arabinosylated form while when overexpressed in the *hpat1-1 hpat2-1* double mutant, arabinosylation was maintained, suggesting that *HPAT3* is primarily responsible for modification of this peptide, and presumably other CLE peptides. However, even within the CLE family, Ara*f*Ts may have substrate preferences. A *Lotus japonicus* putative *HPAT*, *PLENTY* helps regulate the number of nitrogen-fixing root nodules formed as part of the autoregulation of nodulation mechanism (Yoro et al., 2019). Other legume *HPAT*s, i.e., *ROOT DETERMINED NODULATION1* in *Medicago truncatula* and NOD3 in *Pisum sativum* serve the same role (Schnabel et al., 2011). Tri-arabinosylation is required for the nodule suppressing activity of pea CLE peptides, including suppression of the hypernodulation of *nod3* mutants (Hastwell et al., 2019) and in Medicago wild type and *rdn1* mutants. In *L. japonicus*, three CLE peptides (CLE-RS1, 2, and 3) are involved in autoregulation of nodulation (Okamoto et al., 2009; Nishida et al., 2016). Constitutive expression of CLE-RS1 and 2 could suppress the increased root nodule phenotype in *plenty* mutants, but overexpression of CLE-RS3 could not, suggesting that CLE-RS3 is dependent on PLENTY arabinosylation for function, but CLE-RS1 and 2 are not and thus may be arabinosylated by other HPATs (Yoro et al., 2019).

Despite potential target preferences, the Hyp-arabinosylation machinery is remarkably well conserved. Somewhat surprisingly, the phenotypic consequences of its disruption vary dramatically between species. In tomato, loss of the HPAT *fasciated inflorescence* (*fin*) results in severe meristem fasciation due to loss of arabinosylation of *Sl*Clv3. However, in *Arabidopsis*, fasciation does not result from loss of *hpat* activity (MacAlister et al., 2016). Although the fully glycosylated form of Clv3 (Ara*f*_3_-Clv3) has higher activity in *Arabidopsis* than the un-modified form when applied exogenously, the un-arabinosylated form is active (Shinohara and Matsubayashi, 2013; Kim et al., 2017). This difference between tomato and *Arabidopsis* might be a consequence of the selection for larger fruit and, therefore, larger meristems during tomato domestication leaving the size regulation mechanism already near its limit in tomato (Munos et al., 2011). Somewhat surprisingly, the total loss of Hyp-arabinoslyation through mutations of all three *Arabidopsis HPAT* genes is not lethal and does not result in a severe vegetative phenotype (MacAlister et al., 2016). The major phenotype of the triple mutants is a reduction in pollen fertility leading to reduced seed set (Beuder et al., 2020). Similarly, HPAT activity is not essential in the moss *Physcomitrella patens* where knockout of both HPATs resulted in increased biomass due to enhanced elongation of tip-growing vegetative filament cells (MacAlister et al., 2016). In these cases, which Hyp-arabinosylated proteins are responsible for the phenotype is difficult to determine.

### Hyp O-Glycosylation in the Secretory Pathway: Novel Insights and Applicative Opportunities

Large-scale bioinformatics identification of *EXTs* across the plant kingdom indicates that most (76%) encode recognizable signal peptides and are, therefore, likely directed into the conventional secretory pathway (Liu et al., 2016). Signal peptides derived from EXT have been used to direct entry of heterologous proteins into the plant secretory pathway (De Loose et al., 1991; Beuder et al., 2020; Jiang et al., 2020). In addition to a signal peptide, the inclusion of plant *O*-glycosylation motifs can further enhance heterologous protein production in plant cells, *via* an unknown mechanism. Recently, Zhang et al. (2019) demonstrated significantly increased secreted protein yield for enhanced green fluorescence protein (EGFP) when fused to a HypGP module consisting of either 18 tandem repeats of an EXT-like “Ser-Hyp-Hyp-Hyp-Hyp” motif (SP_4_)_18_ or an AGP-like sequence of 32 tandem “Ser-Hyp” repeats (SP)_32_. When expressed in tobacco hairy root culture, the HypGP fused protein was recovered from the culture media at up to 56-fold greater levels compared to an EGFP control lacking a HypGP module. Similarly, Jiang et al. (2020) demonstrated increased secretion and improved solubility of human interferon gamma expressed in *Nicotiana benthamiana* when including a C-terminal AG-type glycomodule, (SP)_10_. The secretion of such HypGP tagged proteins is also highly influenced by nutrient availability, particularly the availability of nitrogen (Zhang et al., 2016). Interestingly, the lack of detectable partially glycosylated intermediates of the (SP_4_)_18_ glycomodule suggests that the rate-limiting step in arabinosylation is either transport to the Golgi or the initiation of arabinosylation with later steps proceeding quickly (Zhang et al., 2019). Work in tomato has demonstrated that the sequentially acting Ara*f*Ts required to modify the *Sl*CLV3 peptide are spatial separated in the *cis*, *medial*, and *trans* Golgi in an order reflecting their order of action (Xu et al., 2015). This suggests a simple linear progression of modification as target proteins move through the Golgi. The observation of the increased rates of Hyp-arabinosylated protein secretion in *hpat1 hpat3* double mutant pollen tubes (Beuder et al., 2020), suggests the existence of either as an active retention mechanism for partially glycosylated species (mutants fail to initiate glycosylation and thus lack the retention signal) or passive retention through the physical interaction between glycosylated proteins and the Ara*f*Ts modifying them until glycosylation is completed. Within the ER, N-linked glycosylation serves as a well-described, conserved mechanism to monitor protein folding, targeting misfolded proteins for degradation (Nagashima et al., 2018; Shenkman and Lederkremer, 2019). How or if glycosylation status is monitored in the Golgi is unclear, however. Unlike the ER, to date, there is no evidence for a general protein glycosylation checkpoint or glycosylation-based quality control mechanism in the Golgi. In animal systems, disrupted Golgi stacking is reported to increase trafficking of several glycoproteins which are released in an under-glycosylated form, suggesting that the rate of glycoprotein secretion is not directly controlled by glycosylation status, but is a consequence of Golgi organization and the accessibility of proteins to the trafficking machinery (Zhang and Wang, 2016). With regard to glycoprotein movement through the secretory pathway, the unanswered questions are numerous and include how or if the rate of trafficking is regulated, the degree to which glycoproteins are selectively chosen for packaging into secretory vesicles, how secretory vesicles are directed to their target membrane, and how glycoprotein secretion relates to secretion of other Golgi products, particularly the carbohydrates destined for the cell wall. Use of synthetic glycosylated reporter proteins holds great promise to answer these and other questions.

## Author Contributions

BP: Hyp-*O*-glycosylation machinery, introduction, discussion, and abstract. PU: HYP-*O*-glycosylated proteins, introduction, discussion, and abstract. CM: discussion: *O*-glycosylation in the secretory pathway, significance of glycosylation on peptide hormones, and plant *O*-glycosylation modules for boosting of glycoprotein production in plants. All authors contributed to the article and approved the submitted version.

### Conflict of Interest

The authors declare that the research was conducted in the absence of any commercial or financial relationships that could be construed as a potential conflict of interest.

## Abbreviations

Hyp: Hydroxyproline
GalT: Galactosyltransferase
PTM: Post translational modification
Ara*f*Ts: Arabinosyltransferase
EXT3: Extensin 3
HRGP: Hydroxyproline-rich glycoprotein
PRPs: Proline-rich proteins
AGPs: Arabinogalactan proteins
LRXs: Leucine-rich repeat extensins
PERKs: proline-rich extensin-like receptor kinases
FH EXTs: Formin-homolog EXTs
CLV3: CLAVATA3
CLE: CLV3/EMBRYO SURROUNDING REGION-related
CEP: C-TERMINALLY ENCODED PEPTIDE
PSY1: PLANT PEPTIDE CONTAINING SULFATED TYROSINE 1
P4H: Prolyl-4-hydroxylase
IDPs: Intrinsically disordered proteins
HypGP: Hydroxyproline glycoprotein
EGFP: Enhanced GFP
